# Left ventricular assist device exchanges: A safe and effective strategy in the era of limited organ availability

**DOI:** 10.1177/03913988251351116

**Published:** 2025-06-25

**Authors:** Nandini Nair, Kenny Nguyen, Dongping Du, Aditya Mahesh, Behzad Soleimani, Balakrishnan Mahesh

**Affiliations:** 1 Division of Cardiology, Penn State College of Medicine, Hershey, PA, USA; 2 Division of Cardiothoracic Surgery, Penn State College of Medicine, Hershey, PA, USA; 3 IMSE, Texas Tech University, Lubbock, TX, USA

**Keywords:** LVAD, pump exchange, surgical approaches to pump exchange, overall survival post pump exchange, long-term survival post pump exchange

## Abstract

**Background:**

Ongoing donor-organ shortage has limited transplantation making LVADs an effective alternative therapy for patients with end-stage heart failure. When LVAD-associated complications arise device exchange is a feasible and safe alternative. This study addresses the factors that impact survival post-LVAD exchange.

**Methods:**

Our decoded database was constructed retrospectively. Surgical details, device features, and re-intervention information were studied. The primary outcome was mortality. Kaplan-Meier estimators were used for post-pump exchange survival analysis. Pairwise log-rank tests compare the survivals between different groups within each variable. *p*-Value <0.05 was considered significant. Backward-stepwise regression was used to construct the multivariable model using a subset of variables, retaining only variables with a *p*-value <0.1. Hazard ratios, their 95% confidence intervals, and p-values of the significant variables were reported.

**Results:**

Analysis of factors impacting survival post-pump exchange study showed a poor survival probability of only primary midline-sternotomy/redo (*p* = 0.005). Multivariable analysis showed that bridging with ECMO was protective with a hazard ratio of 0.16 (0.03–0.86, *p* = 0.03).

**Conclusions:**

The overall survival probability is 50% at 4 years post-pump exchange. This study highlights the differences in post-exchange outcomes depending on the device types and surgical approaches used. LVAD exchange for device-related complications can be performed in high-risk patients as a viable alternative to heart transplantation in the setting of the current heart allocation prioritization systems.

## Introduction

Heart failure (HF) is a global epidemic with a growing population of patients requiring advanced therapeutic interventions. HF results in greater than 1,000,000 hospitalizations and over 600,000 deaths annually making it a leading cause of morbidity and mortality in the United States. Approximately 5% of these patients are ACC/AHA Stage D, requiring advanced surgical therapies such as left ventricular assist devices (LVADs) and heart transplantation. Five thousand heart transplants are performed annually worldwide with >50,000 candidates on the wait list.^1,2^ Ongoing donor-organ shortage has limited transplantation making LVADs an effective alternative either as a bridge-to-transplant (BTT) or as destination therapy (DT). LVADs have sprung into prominence rapidly as a lifesaving option for patients with end-stage HF and have become the standard of care.^3^

Complications of LVAD therapy include infection, pump thrombosis, and equipment malfunction which can be fatal if not treated necessitating LVAD replacement or heart transplantation.^4,5^ Pump thrombosis is a modifiable complication secondary to a complex interplay of device hemocompatibility, blood flow dynamics, and patient specific comorbidities (hypercoagulable states/ suboptimal anticoagulation).^4–6^

The incidence of pump thrombosis in LVADs is device-dependent. HeartMate II (HMII), a second-generation axial-flow pump, demonstrated a notable risk of thrombosis.^7^ Heart Ware (HVAD), a centrifugal-flow device, showed moderate success in thrombosis mitigation, with a 2-year thrombosis rate of 6.4% which was discontinuation due to safety concerns. The 2-year thrombosis rate in HeartMate III (HMIII), a magnetically levitated centrifugal flow pump is 1.1% due to its wider shaft and intrinsic pulsatility.^8^

LVAD exchange is often recommended in the case of pump thrombosis.^4,9,10^ Other important complications requiring exchange include mechanical damage to the driveline (short to shield phenomenon (STS)) and driveline infections. STS occurs in the HMII when a malfunctioning insulator in the driveline cable facilitates a live wire to come into contact with the surrounding metallic shield resulting in massive disruption of power delivery leading to a complete pump stop.^10–12^

Pump exchanges restore hemodynamics and prevent progression to end stage organ failure with favorable outcomes and relatively low postoperative mortality/morbidity when compared to medical therapy.^9^

LVAD exchange may include a complete pump replacement, a partial device upgrade, or driveline exchange. The surgical approach varies depending on patient comorbidities, acuity, device type, and urgency of intervention . Sternotomy and redo-sternotomy have been long-employed techniques that offer direct access to the heart and device components. Less invasive approaches such as sub-xiphoidal and subcostal incisions and right and left anterior lateral thoracotomies have been increasingly adopted to reduce surgical trauma and improve recovery times while limiting infection. When LVAD-associated complications arise multiple studies have shown that minimally invasive techniques for device exchange are both feasible and safe in end stage HF patients.^6,9,13–16^ This study addresses the factors that impact survival post pump exchange.

## Methods

### Patient selection

Thirty-nine patients (48–62 years of age with a median of 56 years) requiring LVAD device exchange from January 2010 to June 2022 were retrospectively assessed. In this cohort (29 males, 74.4%) 28 patients underwent a single LVAD pump exchange, and 11 patients had multiple exchanges constituting 50 patient events. This retrospective study included decoded data from a single center.

### LVADs

Implanted LVADs included (HMII; St. Jude Medical, USA; HMIII; Abbott, Pleasanton, CA), and HVAD (Medtronic Inc, Minneapolis, MN). LVADs included in this study were based on evolution of devices over the past two decades.^17^

### Study design

Our decoded database was constructed retrospectively. LVAD implantation, exchange dates post-implantation/ post-exchange complications, comorbidities, and survival were analyzed. Surgical details, device features, and reintervention information were retrospectively studied. Study endpoints included device explantation, heart transplantation, and death. The primary outcome was mortality.

Institutional review board at the Pennsylvania State University College of Medicine (PSCOM) approved the study. Patient consent was waived as the study was done retrospectively .

### Statistical methods

Time-to-event is the time difference between pump exchange and death or the most recent follow-up. Kaplan-Meier estimators show post-pump exchange survival in different groups. Pairwise log-rank tests compare the survivals between different groups within each variable. *p*-Value <0.05 indicates significance.

Backward-stepwise regression was used to construct the multivariable model using a subset of variables, retaining only variables with a *p*-value <0.1. Hazard ratios, their 95% confidence intervals, and *p*-values of the significant variables were reported.

#### Operative technique reviewed retrospectively

All patients consented before implantation and exchange procedures were included. The surgical approach to LVAD exchange was recorded for each exchange surgery. Echocardiography was used to assess the previously implanted pump position. Implanted pump access was gained mainly via a left subcostal approach often in conjunction with left anterior thoracotomy during LVAD exchange. Other techniques included a redo-median sternotomy or a combination of three approaches to gain access. Complete removal of the previous pump was followed by inspection of the ventricular cavity and the explanted device for thrombi and residual endothelial tissue. The new pump was prepared according to manufacturer’s instructions . Before implantation, every part of the pump was flushed and de-aired. Using standard techniques the driveline was tunneled through the abdominal wall. Once hemostasis was stabilized, a chest tube was placed, and the wound was closed. The anticoagulation regimen included aspirin 81 mg or clopidogrel 75 mg QD in aspirin-intolerant patients, and warfarin to maintain an international normalized ratio of 2.5–3.5.

## Results

Our analysis revealed significant insights into device transition patterns and associated outcomes.

### Conditions requiring pump exchange

**Figure 1.**
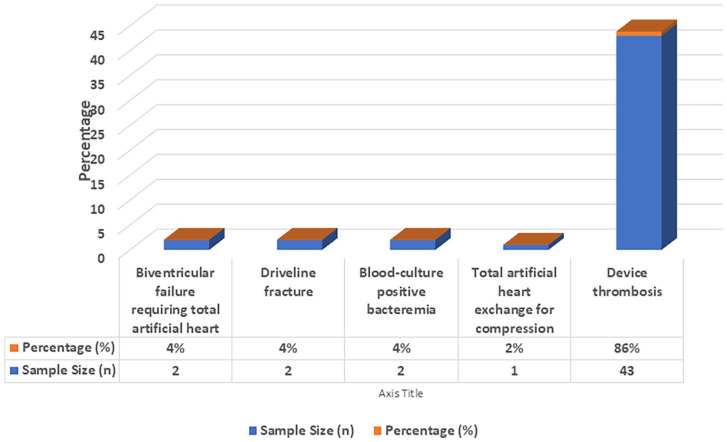
Conditions requiring pump exchange.

Pump exchange procedures were conducted for pump or graft thrombosis in 38 cases (76%), driveline complications in 6 cases (12%), and infection in 6 cases (12%; Figure 1).

### Surgical techniques

**Figure 2.**
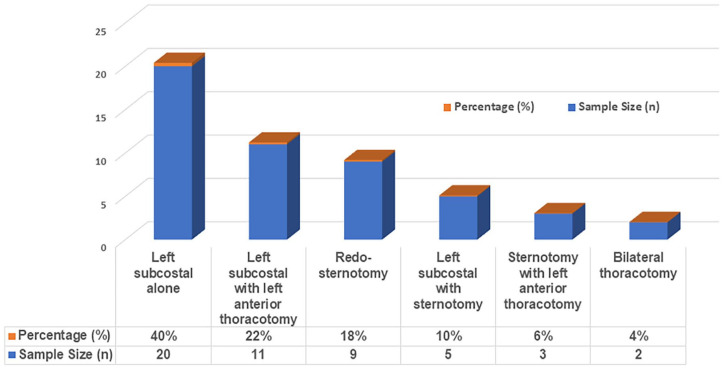
Surgical approaches to LVAD exchanges.

The surgical approaches, their respective sample sizes, and percentages were as follows: Left subcostal only (*n* = 20, 40%), left subcostal with left anterior thoracotomy (*n* = 11, 22%), redo-sternotomy (*n* = 9, 18%), left subcostal with sternotomy (*n* = 5, 10%), sternotomy with left anterior thoracotomy (*n* = 3, 6%), and bilateral thoracotomy (*n* = 2, 4%; Figure 2).

**Figure 3.**
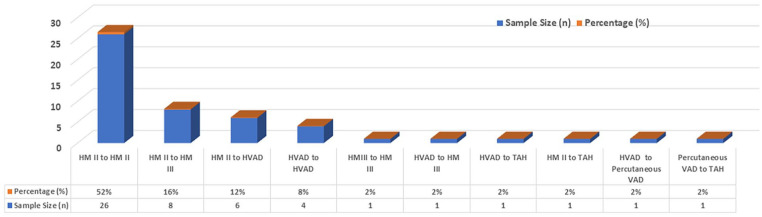
Device types used in LVAD exchanges.

The HMII to HMII formed the majority (*n* = 26, 52%), HMII to HeartWare (*n* = 6, 12%), and HMII to HMIII (*n* = 8, 16%) reflecting the shift toward the newer-generation devices. Intra-device exchanges of HVAD to HVAD (*n* = 4, 8%) and of HMIII to HMIII (*n* = 1). Less frequent but clinically important transitions included HVAD to HMIII, HVAD to Total Artificial Heart (TAH), HMII to TAH, HVAD to Percutaneous Assist Device, and Percutaneous Assist Device to TAH (*n* = 1 in each case, 2%; Figure 3). These findings highlight the evolution of devices over time and the requirement to escalate to biventricular support in a significant proportion of patients based on deterioration of RV function.^17^

Patients requiring an additional pump exchange, 72% (*n* = 8) received a different LVAD. Median survival/follow-up time following additional exchanges was 17.9 months compared to 6.6 months with the first LVAD implantation.

Postoperative outcomes illustrate the risks associated with LVAD exchanges. Reoperation for bleeding was the most common complication (*n* = 8, 16%), ischemic/hemorrhagic stroke (*n* = 3, 6%), RV failure requiring right ventricular assist device (RVAD) implantation (*n* = 2, 4%), major gastrointestinal bleeding (*n* = 1, 2%), and renal impairment requiring long-term hemodialysis (*n* = 1, 2%).

The median follow-up was 20 months (IQR = 3–56 months), during which actuarial survival rates were 72.5 ± 6.5% (1 year) and 39 ± 8% (5 years). These results emphasize the potential benefits/ limitations of LVADs in extending life in patients with advanced HF, particularly with multiple device exchanges. Between exchange events with a sternotomy approach (*n* = 17), medial survival/follow-up time was 17.6 months. Patients with a minimally invasive approach (subcostal, thoracotomy, or a combination of both), *n* = 33, comprised 66% of the study group.

### Analysis of factors impacting survival post pump exchange

**Figure 4.**
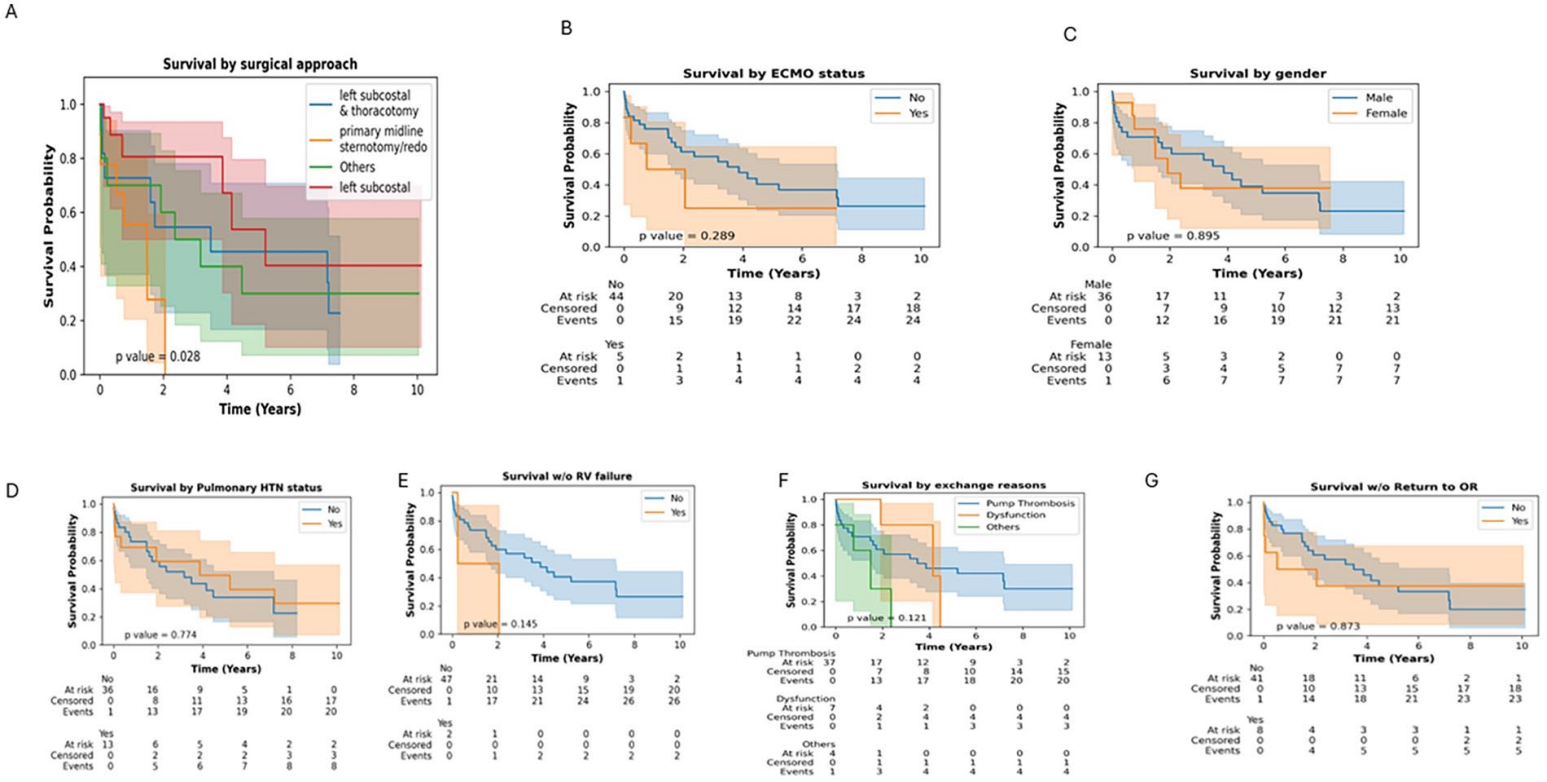
Kaplan-Meir analysis of factors impacting survival post pump exchange. a) Survival by surgical approach, b) Survival by ECMO status c)Survival by gender d)Survival by Pulmonary Hypertension status e)Survival with or without RV failure f)Survival by reasons for exchange g) Survival with or without return to OR

**Figure 5.**
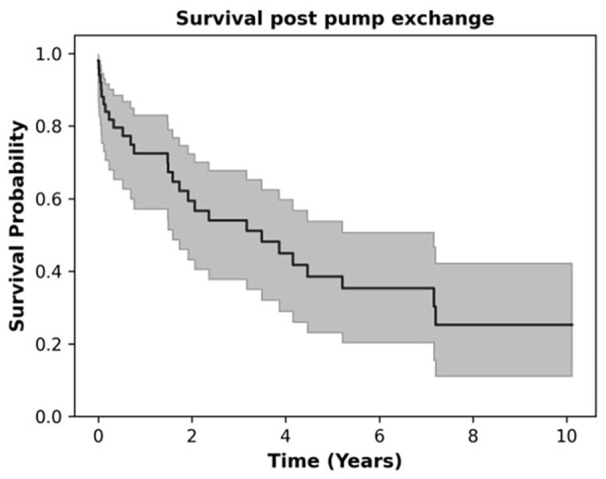
Overall survival post pump exchange.

Figure 4(a) shows that as compared to all the surgical techniques used for pump exchange in this study only primary midline sternotomy/redo showed a poor survival probability (*p* = 0.005). Other risk factors such as ECMO as a bridge-to-decision, gender, pulmonary hypertension, right ventricular failure, reasons for exchange, or return to the operating room did not affect survival probability (Figure 4(b–g) respectively). Figure 5 shows that overall survival probability is 50% at 4 years post pump exchange.

Table 1(a) shows all factors tested while Table 1(b) shows the results of the analysis after removing variables with *p* > 0.1. Bridging with ECMO seems to be protective with HR of 0.16 (0.03–0.86, *p* = 0.03). However, ECMO was a non-significant risk factor on univariate analysis. Pump exchange from HVAD to HVAD carried the highest risk with HR of 19.25 (3.04–122.04, *p* = 0.0017).

**Table 1.** Multivariable analysis.

Variables	Coefficient	Coefficient lower bound	Coefficient upper bound	HR	HR lower bound	HR upper bound	*p* value	*N*
(a)								
Gender_1	1.00	−0.52	2.53	2.73	0.60	12.51	0.196	36
ECMO_Yes	−1.67	−3.60	0.26	0.19	0.03	1.30	0.091	6
HTN_1	−0.22	−1.21	0.77	0.80	0.30	2.16	0.663	13
OR_1	−0.59	−1.95	0.77	0.55	0.14	2.16	0.394	8
Exchange Type_HM II to HM II	1.96	−0.40	4.32	7.08	0.67	74.99	0.104	26
Exchange Type_HM II to HVAD	1.05	−0.53	2.62	2.85	0.59	13.76	0.192	5
Exchange Type_HVAD to HVAD	5.22	1.97	8.48	185.46	7.14	4818.60	0.002*	4
Exchange Type_Other	2.87	0.11	5.63	17.58	1.11	277.99	0.042	7
Driveline dysfunction (fracture/electrical failure, short to short/infection)^a^	−0.11	−1.51	1.29	0.90	0.22	3.63	0.877	7
Everything else/other = 3^a^	2.04	−0.20	4.29	7.73	0.82	73.14	0.075	5
Left subcostal + thoracotomy^b^	1.79	−0.60	4.18	5.98	0.55	65.38	0.143	11
Primary midline sternotomy/redo only^b^	0.90	−1.08	2.88	2.45	0.34	17.75	0.375	9
Everything else/other, which include^b^								
Left subcostal + sternotomy, 4 = bilateral thoracotomy/only, and 6 = sternotomy + thoracotomy	0.42	−1.04	1.88	1.52	0.35	6.53	0.576	10
7 subcostal + sternotomy (3, 4, 6, and 7 were grouped into “other” because they have small patient numbers)^b^								
Concomitant other cardiac procedures: yes as compared to No	1.26	−0.63	3.14	3.52	0.53	23.12	0.191	5
The exchange reason key is								
As compared to pump thrombosis = 1 (baseline)^a^								
Surgical approach key is								
As compared to 1 = left subcostal only (baseline)^b^								
(b)								
ECMO_1	−1.85	−3.55	−0.15	0.16	0.03	0.86	0.0332	6
Exchange Type_HM II to HVAD	1.09	0.04	2.14	2.97	1.04	8.47	0.0417	5
Exchange Type_HVAD to HVAD	2.96	1.11	4.80	19.25	3.04	122.04	0.0017	4
Exchange Type_Other	1.35	0.08	2.62	3.87	1.09	13.80	0.0366	7
Other exchanges	1.49	0.14	2.83	4.43	1.16	16.98	0.0300	5
Concomitant cardiac procedures	1.42	−0.08	2.92	4.14	0.92	18.60	0.0640	5

a All factors tested

b Factors tested after removing variables with *p* > 0.1

*p* < 0.05*

## Discussion

LVADs have transitioned from a short-term BTT strategy into long-term durable devices with capabilities to extend life. This shift reflects extended heart transplant waiting times in the current allocation system and the growing adoption of LVADs as destination therapy in increasing number of patients. Previous studies have outlined techniques required for exchanging left ventricular assist devices, but these studies involved using older devices no longer used or are being phased out.^14–18^ Our study presents survival, and complications in patients requiring LVAD exchanges in the current era . Despite the higher risk profile of patients, LVAD exchange remains a viable alternative to orthotopic heart transplantation in the current organ allocation system.

### Surgical approach

The approach to LVAD placement is determined by the patient’s mediastinal anatomy/characteristics, which influences the subsequent exchange operation outcomes. Although contemporary devices (HMII, HVAD, and HMIII)have modular designs, the components are incompatible across various devices. Therefore, LVAD exchanges involving upgrades or replacements often require maximal exposure with a sternotomy approach. New developments include minimally invasive approaches. Intuitively, limiting the size and number of incisions should be associated with decreased morbidity due to less dissection of the re-operative mediastinum, however, the selection is more nuanced contingent on the indication for surgery. A major indication for pump exchange is device thrombosis occurring in two thirds of patients because pump exchange has superior survival compared to thrombolysis and lower recurrence.^10^

Current literature suggests lower morbidity with minimally invasive approaches with limited incisions versus redo sternotomy especially in pathologies involving outflow grafts and those limited to the pump/driveline suggesting non-sternotomy approaches, off cardiopulmonary bypass, for benefits of minimally invasive strategies.^14,19–21^ Our surgical approaches comprised of left subcostal, left subcostal with left anterior thoracotomy, redo-sternotomy, left subcostal with sternotomy, sternotomy with left anterior thoracotomy, and bilateral thoracotomy, consistent with previous studies. At our center and in others, the lateral thoracotomy approach has been shown to be associated with higher short-term survival, reduced incidence of RV failure, lower postoperative RVAD need, shorter ICU stay, less bleeding requiring transfusions, and reduced incidence of renal failure but no difference in stroke rates.^16,22,23^ The subcostal approach also appears to be a favorable alternative to sternotomy, with reduction in operative time, cardiopulmonary bypass time, blood transfusions, incidence of prolonged intubation, tracheostomy, and acute kidney injury requiring dialysis.^16^ HeartMate II device exchange by a subcostal approach in a single-center retrospective study showed 0% in-hospital mortality while the sternotomy group had 17% which was not significant due to a small sample size.^24^

Patient outcomes to the different surgical approaches in our study are consistent with those reported in the current literature. Our patients primarily underwent minimally invasive approaches when a redo-sternotomy could be avoided though variability between health systems may still impact outcomes.

Redo sternotomy becomes necessary for extensive infection/thrombosis, concomitant intracardiac procedure and upgrade with incompatible devices. Our current and previous studies show that compared to all the surgical techniques for pump exchange midline redo-sternotomy has the worst survival probability possibly due to pathology necessitating complete LVAD exchange including the infected outflow graft.^16^ Patients with infected LVADs generally tend to be septic and dependent on vasoconstrictors for maintenance of blood pressure. Infection in the LVAD is associated with worse RV dysfunction after complete pump exchange. Extensive dissection and longer time on cardiopulmonary bypass required for complete pump exchange necessitates administration of multiple transfusion with red blood cells, platelets, and procoagulant factors for correcting the ensuing coagulopathy which predispose patients toward longer periods on the ventilator requiring continuous renal replacement therapy which leads to increased RV dysfunction, sternal complications and higher perioperative mortality following a complete pump exchange.^16,19,20^

Ideal treatment for LVAD related complications remains heart transplantation but associated comorbidities preclude transplant in many patients making pump exchange a life-saving alternative.^21^ Cardiac transplantation for pump infection, predisposes to wound infections including osteomyelitis which can be life-threatening.^21^

### Complications

Of all LVAD exchange operation complications (bleeding, strokes, risk of re-thrombosis, postoperative dialysis), RV failure remains most significant complication.^25^ Our cohort did not experience extensive right HF requiring RVAD support as reported especially in HVADs.^26^ Device exchange can be performed with low early mortality and optimal late survival.^9,16,25,27^ Device-related infection requiring exchange if performed within 4 months of diagnosis, showed freedom from recurrence of infection of 96% versus 56% in exchanges at >4 months post diagnosis. However, another study showed device exchange for LVAD infection had worse outcomes than exchange for device malfunction/thrombosis, requiring expeditious management for better survival.^17,28^

Difference between surgical device exchange in comparison to medical therapy in treating CF-LVAD thrombosis in 43 patients (28 requiring exchange, 1 explant, and 14 with medical therapy), the exchange group had significantly lower in-hospital mortality and higher 1-year survival and freedom from cerebrovascular accidents.^29^ An increase in 1-year survival when comparing exchange to non-exchange patients, a survival rate that is non-inferior to conservative management, and comparable postoperative mortality at 30 days between LVAD exchange and primary implantation have been reported.^10,30^ Repeated LVAD exchanges, in our study, showed results consistent with that reported of an overall survival of 72% in 1 year and 60% at 2 years.^31^ In some studies HMIII LVADs upgraded during an exchange showed lower stroke incidence and higher survival than the HVAD. Other studies show complicated clinical courses when exchanging from HVAD to HMIII versus HMII to HMIII.^13,32^ Three-year follow-up after LVAD exchange to HMIII demonstrated superior hemocompatibility for pump thrombosis.^33^ HVAD to HMIII exchange showed a lower survival as compared to those remaining on continued HVAD support . HVAD to HVAD exchange had a lower survival than HVAD to HMIII exchange.^34^ Although LVAD patients who are refractory to medical therapy require device exchanges, the cost, antithrombotic therapy, and potential impact on heart transplant graft survival due to allosensitization in BTT patients must be considered.^35,36^ Device exchange between different device types involves complex decision-making and risk versus benefit evaluation to tailor to individual scenarios to deliver care in a safe and personalized fashion.^37^

### Factors affecting survival were analyzed by univariate and multivariate analysis

The protection noted in patients bridged with ECMO in multivariate analysis may be explained as its effect as a bridge on improving perfusion/ relieving multiorgan failure creating a pathway to improve outcomes post LVAD exchange or due to multivariate interactions in a small cohort. This finding will need further validation in large studies.

Although a small retrospective single-center study, this paper presents a unique analysis to identify risk factors for long-term survival in the post LVAD exchange population and its role in extending life significantly in an era of organ shortage.

## Limitations

This was a retrospective analysis conducted in a single academic institution with low event numbers. Management of complications, surgeon-specific technical skills, patient comorbidities, and acuity may not be applicable across different institutions. The impact of the surgical approach on patient outcomes need validation large multicenter studies.

## Conclusion

LVAD exchange for device-related complications can be performed safely and effectively in high-risk patients and is a viable alternative to heart transplantation in the setting of the current heart allocation prioritization systems. Compared to all the surgical techniques used for pump exchange in this study only primary midline sternotomy/redo showed a significant poor survival probability. The overall survival probability is 50% at 4 years post pump exchange. This study highlights the differences in post exchange outcomes depending on the device types and surgical approaches used.
